# Endemic fish species structuring oceanic intertidal reef assemblages

**DOI:** 10.1038/s41598-018-29088-0

**Published:** 2018-07-17

**Authors:** Ryan Andrades, José Amorim Reis-Filho, Raphael M. Macieira, Tommaso Giarrizzo, Jean-Christophe Joyeux

**Affiliations:** 1 Laboratório de Ictiologia, Departamento de Oceanografia e Ecologia, Universidade Federal do Espírito Santo, Av. Fernando Ferrari, 514, Goiabeiras, Vitória, Espírito Santo 29075-910 Brazil; 2 Instituto de Biologia, Universidade Federal da Bahia, Campus Ondina, Salvador, Bahia 40170-115 Brazil; 3 Laboratório de Ecologia Marinha, Universidade Vila Velha, Rua Comissário José Dantas de Melo, 21, Boa Vista, Vila Velha, Espírito Santo 29102-770 Brazil; 4 Laboratório de Biologia Pesqueira e Manejo dos Recursos Aquáticos, Universidade Federal do Pará, Av. Perimetral, 2651, Terra Firme, Belém, 66075-110 Brazil

## Abstract

Intertidal reef environments are facing a global crisis as climate changes are causing sea-level rise. Synergistically, other human-induced impacts (e.g., sewage, habitat loss) caused by concentration of human populations near the coast increase the natural vulnerability of intertidal ecosystems. However, the effect of these threats have long been neglected due, in part, to a limited knowledge of some aspects of intertidal fish ecology. We tested what are the main differences and drivers in fish assemblages structure between tidepools in three oceanic and three continental shelf (coastal) sites of the tropical southwestern Atlantic (Brazilian Province) using standardized sampling methods. Oceanic and coastal fish assemblages were distinctly structured at the trophic and composition levels. The noteworthy endemism species rate (38–44%) and high densities in oceanic sites are supported by resident species restricted to mid and high-shore tidepools where herbivores were the major trophic group. The coastal sites, on the other hand, were dominated by widely distributed and carnivore species. Physical (substrate type, pool height, subtidal distance and rock coverage), biological (sessile animal coverage) and chemical (salinity) parameters act as the driving forces influencing fish spatial occupancy. Here, clear evidences of high fish endemism and importance of endemics structuring oceanic communities may act as the last straw in favor of the conservation of oceanic intertidal reefs.

## Introduction

An extraordinary characteristic of the marine life is its high diversity estimated above one million species^1^. However, indiscriminate use of marine ecosystems and resources has been promoting changes in biota^2–5^, massively impacting communities structure and ecosystem services^5–7^. In this context, reef environments (e.g., coral and rocky reefs) are pointed out as a priority for conservation planning (MPAs - marine protected areas as example of effort) to ensure support to biodiversity and reef services such as fish stocks maintenance, carbon uptake, tourism and shoreline protection^8,9^.

Intertidal reef habitats are located at the land-ocean interface and provide the same essential ecological services than other reef types^10^. Surprisingly, they have been neglected or dismissed in discussions involving management and conservation planning to solve, or at least mitigate, the reef environmental crisis^5,11–13^, even while recognizing that these ecosystems probably are among the most impacted in the marine realm^5,13^. Their vulnerability arises from easy access, proximity of urban areas, intensive use in tourism, harvesting activities and sea level rise. In intertidal fishes, compounding factors include characteristically short displacements by small-sized species with strong site fidelity and specialized habitat requirements^14,15^ and use as nursery grounds for ecologically and commercially important species^16–19^, replenishing adult populations in subtidal habitats^18,20^. These organisms also present diverse life strategies, living all (i.e., permanent residents) or part of their life cycle (i.e., secondary residents and transient species) in tidepools^21,22^.

The structural complexity, partial isolation and species richness of tidepools can be used as proxies to assess a variety of ecological and anthropic impacts scenarios to gain a better understanding of marine systems changes over time^10,23,24^. Intertidal environments are particularly good models because of the possibility to measure community metrics (i.e., density, number of taxa or biomass) using actual tridimensional data (volume). Among other benefits is the efficient record of all taxa, including small-sized and cryptobenthic fish species. These are important links between lower and higher trophic levels^25^, but are largely underestimated in standard subtidal fish samplings (e.g., visual census^26^).

During its isolation from the sea, tidepool water greatly varies in temperature, salinity, pH and dissolved oxygen, and these variation are closely related to pool characteristics such as height in relation to sea level, substrate nature, benthic cover, superficial area, and volume^10^. The combination between water variables and pool characteristics strongly influences the distribution of fish species in the intertidal space and the fish assemblage structure in each tidepool^19,27,28^. In tropical habitats, a most-important role in structuring fish assemblages is expected to be played by biotic interactions while in temperate habitats the wider fluctuations in abiotic conditions would structure assemblages seasonally^29^. However, the distribution patterns of tropical tidepool fishes may be dependent of substrate characteristics^30^ and sub-tropical and temperate tidepool fishes may present diel variations and variability in habitat use to avoid intra and inter-specific competition and predation^27,31–33^. These contrasting findings point out the urgent necessity to understand how interactions between biotic and abiotic factors structure fish assemblages.

In comparison to coast, oceanic environments had received little attention due to isolation and logistic challenges. However, the number of island studies has been growing substantially during the last decades, with endemism emerging as a key feature shaping island community ecology^34–37^. While some progress has been made, many aspects of oceanic intertidal fishes (e.g., evolution, systematics and ecology) remain poorly known^16,18,19,38^. Thus, we use intertidal fishes in oceanic and coastal tidepools to test whether taxonomic and trophic assemblages structure differs between regions, determining the importance of habitat features as structuring drivers on these assemblages. Additionally, we evidence the structuring role of endemic species in oceanic communities and provide new data for tidepool fish assemblages in two non-studied MPAs in the South Atlantic, the only atoll in the South Atlantic (Rocas Atoll) and Fernando de Noronha Archipelago.

## Results

### Fish assemblage structure

**Figure 1:**
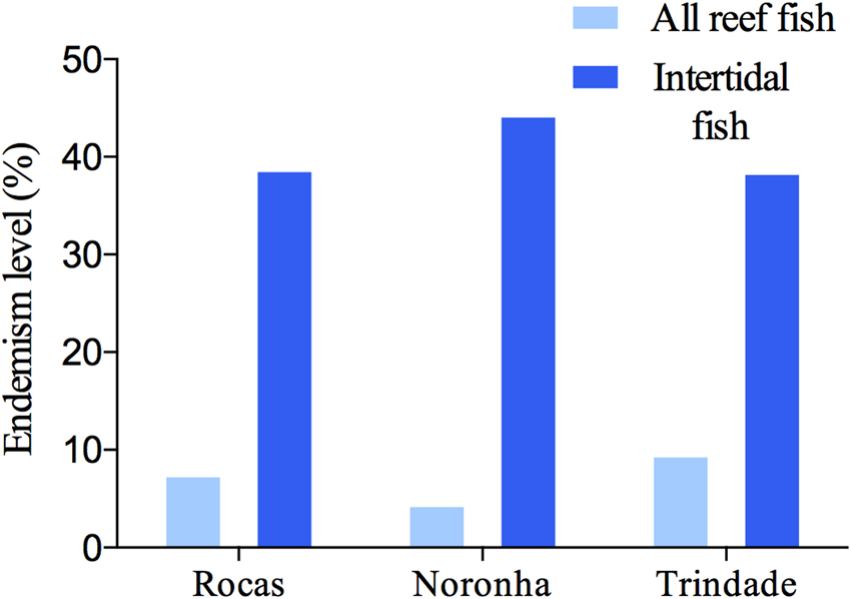
Fish endemism levels at oceanic sites for all reef fish fauna^40,42,73^ and for intertidal fishes (present work).

Fifty-nine taxa belonging to 21 families were sampled, 28 in the coastal region (Salinópolis, Jericoacoara and Anchieta) and 35 in the oceanic region (Rocas, Noronha and Trindade) (Supplementary Table S1). Endemism levels were noteworthy in oceanic sites, 39% (7 of 18 species) for Rocas, 44% (8 of 18 species) for Noronha and 38% (5 of 13 species) for Trindade and remarkably higher than the total reef fish endemism including all reef fish species present in the subtidal and intertidal zones (Fig. 1). In coastal sites endemism was low, 18% in Salinópolis, 17% in Jericoacoara and 18% in Anchieta.

**Figure 2:**
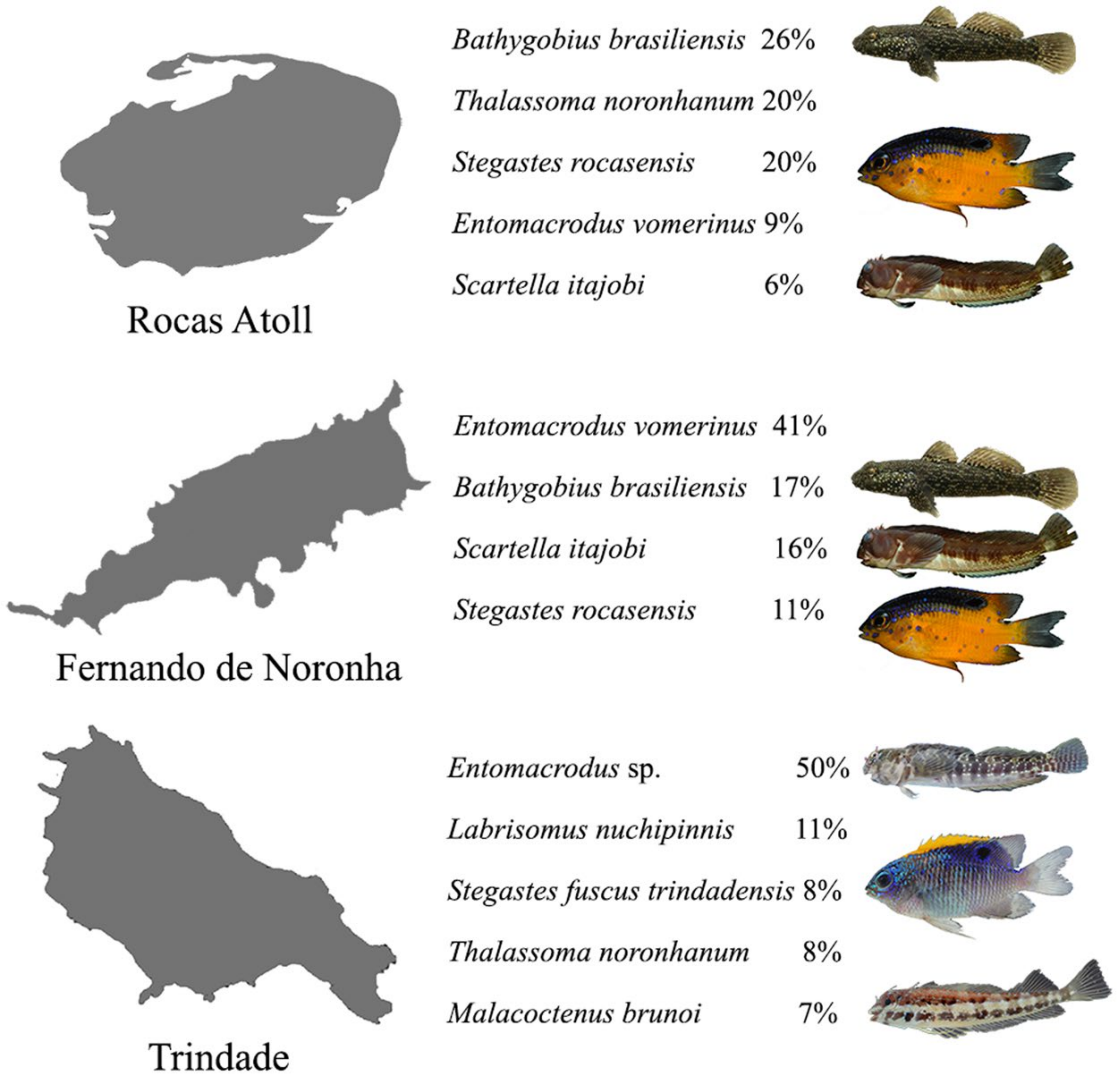
Species contribution to assemblage similarity percentage (>80%) in density for each island. Endemic species (pictured) are *Bathygobius brasiliensis*, *Stegastes rocasensis* and *Scartella itajobi* in Rocas Atoll, *B. brasiliensis*, *S. itajobi* and *S. rocasensis* in Noronha and *Entomacrodus* sp., *Stegastes fuscus trindadensis* and *Malacoctenus brunoi* in Trindade. Map data ©2017 Google, edited and assembled in CorelDraw X5. Fish photos: R. Andrades, R. M. Macieira and J.-C. Joyeux.

**Figure 3:**
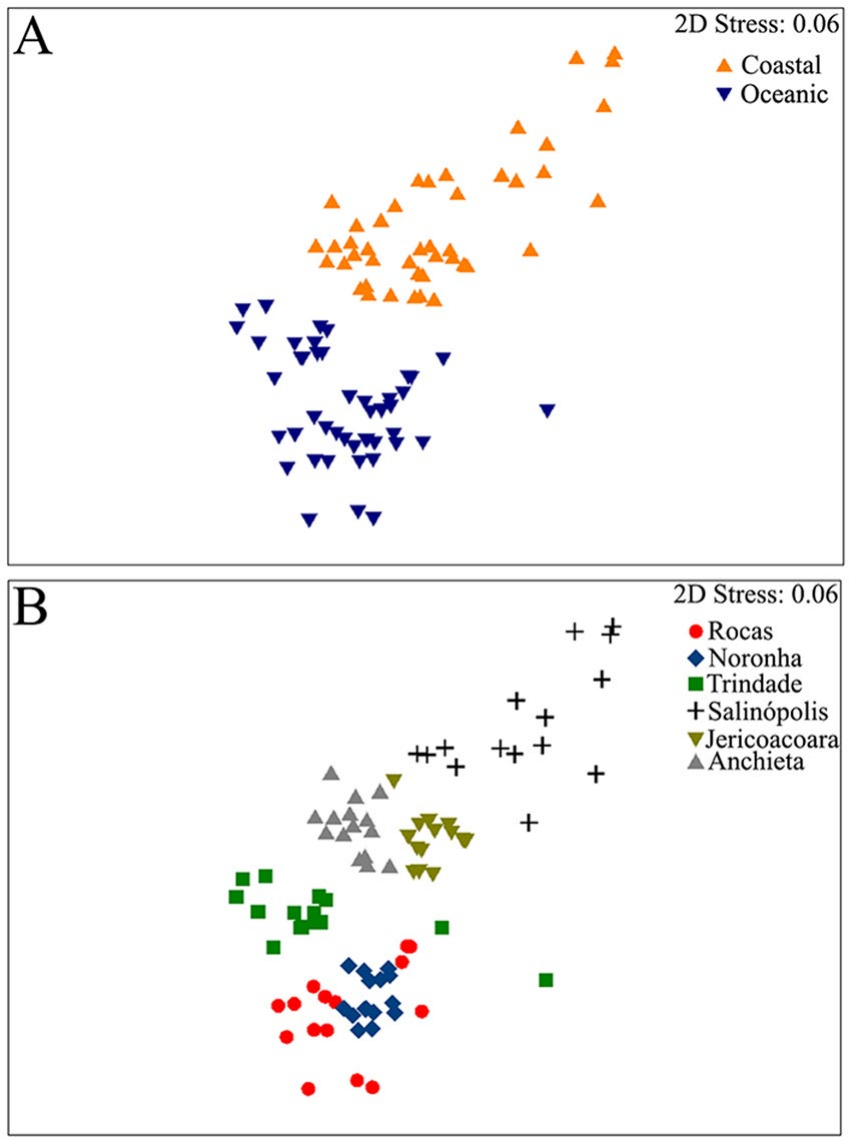
Multidimensional scaling using fish density data in tidepools (N = 90) in regions (**A**) and sites (**B**).

Fish assemblages differed significantly between coastal and oceanic regions (PERMANOVA F = 2.3; MS = 67,104; *p* < 0.05) and among sites (PERMANOVA F = 16.5; MS = 29,219; *p* < 0.05). In addition, SIMPER analysis evidenced that endemic species strongly structured intertidal assemblages in oceanic sites (Fig. 2). In contrast, in coastal sites widespread species contributed more to fish assemblages albeit some Brazilian coastal endemics seem to be locally relevant (e.g., *Barbulifer* sp. and *Gobiosoma* sp. in Salinópolis; Supplementary Table S2). As expected from PERMANOVA results, nMDS ordination segregated pools between regions (Fig. 3A) and, not as distinctively, among sites within regions (Fig. 3B).

**Figure 4:**
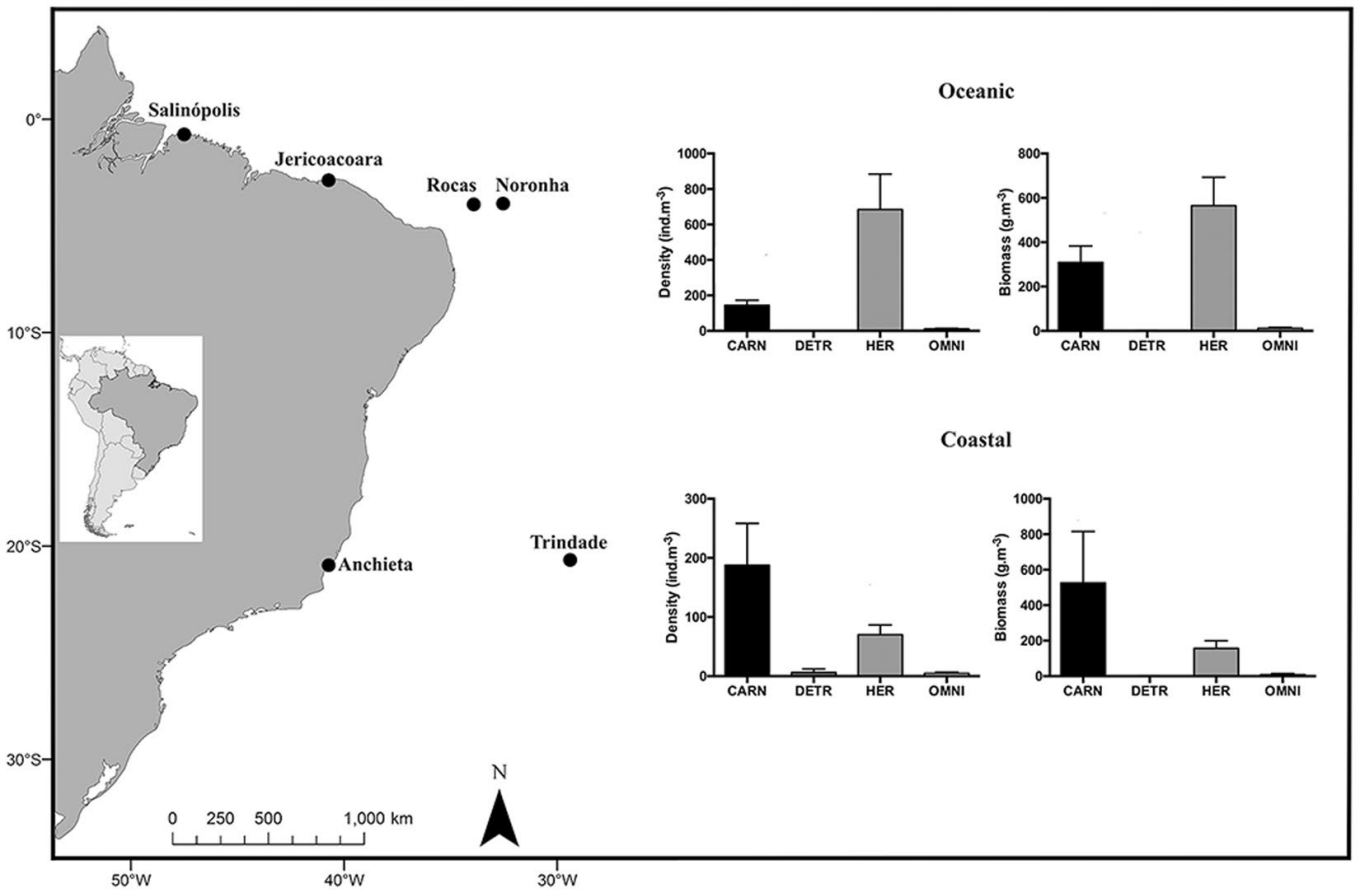
Study sites in the southwestern Atlantic (left) and mean (+SE) fish density and biomass of the main trophic groups (CARN = carnivores, DETR = detritivores, HER = herbivores and OMNI = omnivores) in oceanic and coastal regions. Map data ©2017 Google, edited and assembled in CorelDraw X5.

Predominance of trophic groups was dissimilar between oceanic and coastal regions (ANOSIM Global R = 0.2; *p* < 0.01) with prevalence of herbivores in oceanic habitats and carnivores in coastal ones. In fact, trophic groups differed in density and biomass between both regions (Kruskall-Wallis; *p* < 0.05; Fig. 4). The two main trophic groups (carnivores and herbivores) also differed significantly within each region (Mann-Whitney; *p* < 0.05), except for biomass in the coastal region. When trophic groups were segregated into subcategories (feeding habits), territorial herbivores and mobile invertebrate feeders were predominant among herbivores and carnivores, respectively.

### Patterns of tidepool species use

**Figure 5:**
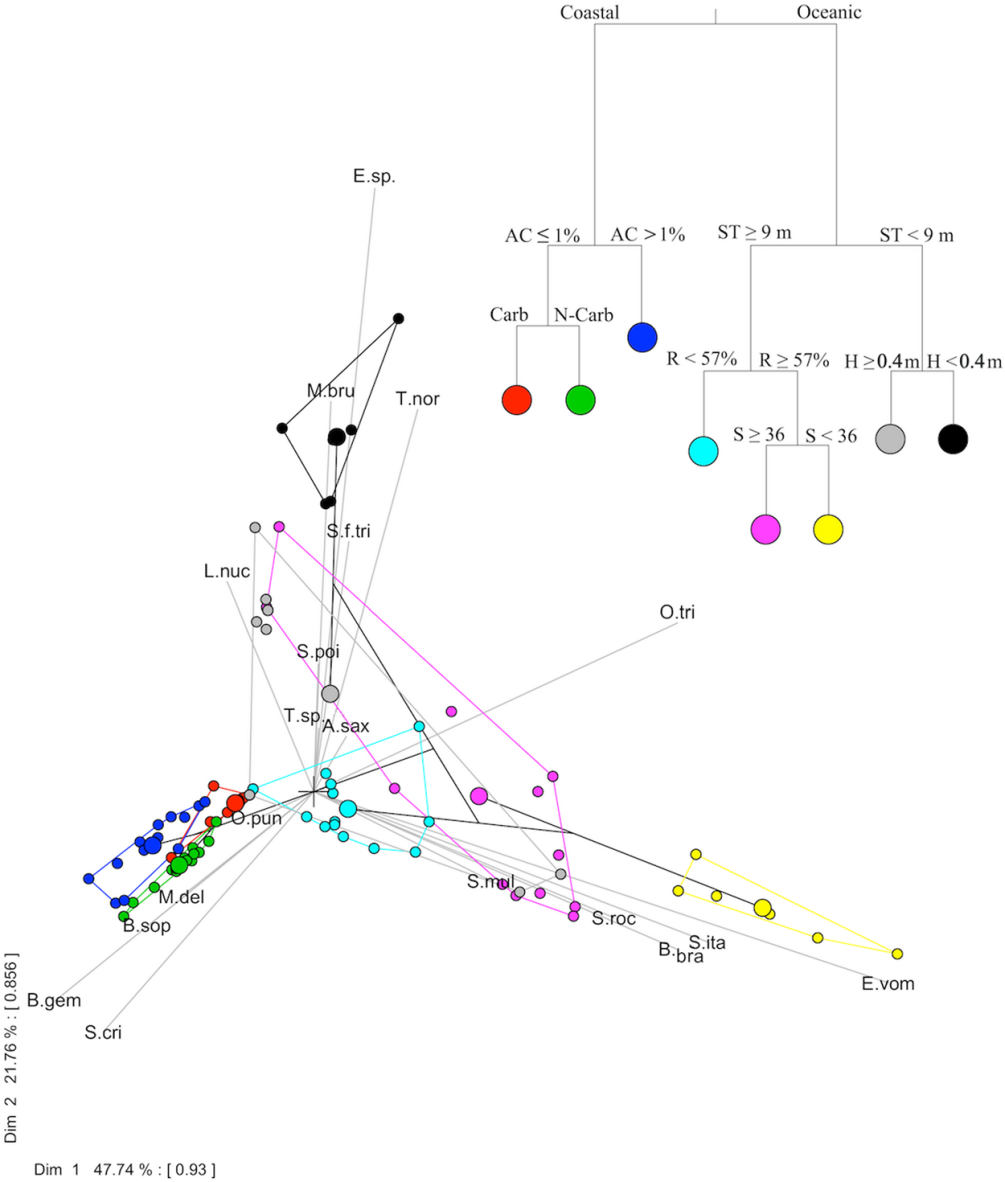
Multivariate regression tree (MRT) analysis with the tree (top right) and principal component analysis (PCA) diagram for the tidepool fish species and environmental explanatory variables (bottom left). Dot color in PCA refers to MRT leaf color. MRT explanatory variables are Region (Oceanic and Coastal), Sessile animal coverage (AC), Subtidal distance (ST), Substrate (Carbonate = Carb and Non-carbonate = N-Carb), Rocky coverage (R), Pool height (H) and Salinity (S). Fish species shown in PCA are *Abudefduf saxatilis* (A.sax), *Bathygobius brasiliensis* (B.bra), *Bathygobius* s*oporator* (B.sop), *Bathygobius geminatus* (B.gem), *Entomacrodus* sp. (E.sp.), *Entomacrodus vomerinus* (E.vom), *Labrisomus nuchipinnis* (L.nuc), *Malacoctenus delalandii* (M.del), *Malacoctenus brunoi* (M.bru), *Omobranchus punctatus* (O.pun), *Ophioblennius trinitatis* (O.tri), *Scartella cristata* (S.cri), *Scartella itajobi* (S.ita), *Scartella poiti* (S.poi), *Starksia multilepis* (S.mul), *Stegastes fuscus trindadensis* (S.f.tri), *Stegastes rocasensis* (S.roc), *Thalassoma noronhanum* (T.nor) and *Tomicodon* sp. (T.sp.).

Seven explanatory variables structured intertidal fish fauna: Region, Sessile animal coverage, Subtidal distance, Substrate, Rock coverage, Pool height and Salinity (Multivariate Regression Tree-MRT; Fig. 5). Variance explained by the tree was 56%; individual splits explained 18% (Region), 10% (Animal coverage), 6% (Subtidal distance), 6% (Substrate), 6% (Rock coverage), 5% (Pool height) and 5% (Salinity). MRT error was low (0.443), cross-validated relative error was 0.726 and standard error 0.075. The first two principal component analysis axes accounted for 47.74 and 21.76 of between-groups sums of squares. Axes interset correlations were high (0.93 and 0.86), which confirmed the strong species-environment variables relationship, including for endemic taxa. Variables related to substrate (substrate type and rock coverage) influenced both oceanic and coastal fish assemblages. Tidepool position (subtidal distance and height) and sessile or slow-moving fauna were important for, respectively, oceanic and coastal assemblages.

## Discussion

Endemism and herbivory are key factors that guide composition and trophic structure of oceanic intertidal fish assemblages in southwestern Atlantic (SWA) islands. This contrasts with previous findings on SWA reef fishes, mainly based on studies at Rocas, in which assemblage structure in oceanic and coastal sites differ due to high density of planktivorous fishes at islands^39,40^. This can be ascribed to the centimeters-deep tidepool water layer which probably offers neither space nor feeding resource to planktivores such as *Chromis multilineata* and *Myripristis jacobus*. However, these are common on subtidal reefs at the studied sites^41,42^, and it is reasonable to infer that they may visit the intertidal reefs during high tides. Their rarity or absence in tidepools at islands and elsewhere^43,44^ may allow other trophic groups to take the lead. This idea is supported by recent evidence of herbivorous species steadily acting as omnivores in the intertidal zone^45^. Changes in environment conditions exist and may impose exceedingly steep adaptive gradients to biota. For example, the higher feeding pressure of herbivores in oceanic islands, compared to coastal sites, was related to high density and biomass of territorial herbivores, *Entomacrodus* species and *Ophioblennius trinitatis*. The evolution of these combtooth blennies in intertidal oceanic ecotones may be explained by an adaptive set of physiological, morphological and behavioral traits to life under intertidal harsh conditions (e.g., high temperature and salinity changes, wave impact and desiccation risk)^19,38,46,47^. In addition, this family comprises many amphibious species^48^. According to recent findings^48^, repeated evolutionary development of amphibious behavior in fish is directly related to evolution in novel environments. The intertidal zone offers optimal conditions to expose fishes to a dual, aquatic and terrestrial, life through the oscillation of tides. The rockskippers *Entomacrodus* along many Blenniidae and a number of Gobiidae can emerge from water under abiotic (e.g., water temperature, dissolved oxygen and pH) or biotic pressure (e.g., competition or predation), thriving or at least surviving on dry grounds during a significant period of time if necessary^49,50^. Under these conditions, fishes may avoid dehydration using crevices or under algae^15,51,52^, although desiccation cannot always be avoided^47^.

However, adaptation in oceanic islands is more complex than in contiguous, mainland habitats, requiring self-recruitment that is partly dependent upon island features such as isolation, area, age and sea-level fluctuations^53^. In our study, intertidal gobiids are absent from the most isolated island, Trindade^18^, including the intertidal/shallow-water, widespread and iconic frillfin gobies *Bathygobius*. Several species of the family, none intertidal, are present in subtidal reefs^54^. While *Bathygobius* short pelagic larval duration (PLD) may limit long-distance dispersal from the mainland 1,200 km away (but probably much less during the Pleistocene), long PLDs (49 days and over 1,000 km in *Ophioblennius atlanticus*^55^) in Blenniidae may partly explain the presence of *Entomacrodus* sp. and *Ophioblennius trinitatis* in all Brazilian islands. In contrast, the permanency of *Tomicodon* sp. in Trindade, with Gobiesocidae having shorter PLDs than Gobiidae^56^, would be assured by self-recruitment. Pinheiro *et al*.^53^ stated that the turn-over of weak dispersers (e.g., *Bathygobius, Tomicodon)* in evolutionary time-scales is common in oceanic islands. It is reasonable to suggest that, considering the balance of low immigration and high extinction rates, many species (e.g., *Bathygobius*) could have colonized Trindade in the past to become extinct at some later time.

Our data indicate that intertidal fish assemblages in oceanic islands of the southwestern Atlantic are mainly structured by endemic reef fish species. Direct comparisons of most abundant species in subtidal and intertidal habitats also clearly evidenced the importance of endemics in the intertidal. For instance, in Trindade the most abundant species in subtidal habitats are non-endemics *Cephalopholis fulva*, *Melichthys niger* and *Thalassoma noronhanum*^42^, whereas in tidepools the endemics *Entomacrodus* sp. and *Malacoctenus brunoi* are two of the three most abundant species. A variety of adaptations to live in intertidal waters was detected in endemic taxa, with niche habitat requirements differing between oceanic sites, while distinct environmental variables act as drivers for oceanic and coastal fish species. In Rocas and Noronha, endemic *Bathygobius brasiliensis* and *Scartella itajobi* were associated to tidepool salinity while in Trindade distribution of endemic *Entomacrodus* sp. and *Malacoctenus brunoi* is dependent upon tidepool height. Thus, the fish assemblages we studied obey neutral theory, as evidenced by differences among sites within regions, and niche theory. The latter is strongly supported by between-regions differences in trophic structure pattern and endemic species importance that suggest island-habitat filtering.

Despite their noticeable importance and the fact that tidepools are one of the easiest marine ecosystems to access and study in oceanic islands, they remain neglected. For instance, there is only one published work on tidepool fish assemblage structure in any Brazilian oceanic islands^18^, compared to a number of works on subtidal reef fish assemblages at the same sites^41,42,57,58^. Tidepool resident often are small-sized species inhabiting small territories and restricted to rocky shores. Such species are highly vulnerable to local impacts such as oil spills, commercial exploitation and recreational activities. Endemic populations in oceanic islands are especially at risk since their extent of occurrence is restricted to a shallow fringe around the islands. Logistics involved in monitoring, if not creating, marine protected areas of -or starting at- intertidal habitats are straightforward and probably relatively cheap in comparison to other marine habitats. High fish endemism in island intertidal reef environments is an ubiquitous pattern worldwide^13,27^ reaching over to 60% in Australia and New Zealand^22,59,60^. Sea-level rise associated with climate changes and local anthropogenic impacts (e.g., sewage, reef trampling and organisms removal) induce profound changes and may lead to depletion and extinction of endemic populations. Future reef conservation planning must consider intertidal reef areas as one of most vulnerable reef habitats^13^. We hope this study puts into perspective the urgency in considering intertidal habitats as a priority in conservation planning, management and restoration before their fish populations are suppressed by local and global threats.

## Methods

### Study areas

Tropical intertidal habitats of six southwestern Atlantic sites of the Brazilian Province were sampled for fish assemblages, three in oceanic environments, Rocas Atoll (03°51′S; 33°49′W), Fernando de Noronha Archipelago (03°50′S; 32°25′W) and Trindade Island (20°30′S; 29°20′W), and three in coastal environments of the continental shelf, Salinópolis (00°36′S; 47°21′W), Jericoacoara (02°47′S; 40°30′W) and Anchieta (20°49′S; 40°36′W) (Fig. 1). Briefly, tidepools are mostly situated in moderately flat areas built primarily by phonolite rocks or biogenic carbonate substrate in oceanic sites and biogenic carbonate or beach rocks in coastal sites (Supplementary Fig. S1). Further environmental data are provided in Supplementary Information.

### Field sampling

Fifteen tidepools were sampled in each site and all field expeditions were performed during the dry season periods of 2014 (Rocas Atoll) and 2015 (other sites). Tidepools were chosen at random during the ebb tide providing there was no connectivity to the sea or to other pools. A set of environmental variables was recorded for each pool sampled. Pool water temperature, salinity and pH were measured right before sampling with a digital thermometer (0.1 degree precision), refractometer (1 psu) and pH meter (0.1 unit), respectively. Measurements (bathymetry and topography) were made immediately after water characterization using a 10 × 10 cm grid with depth measured at each intersection point. Pool surface area (water surface), planar area (bottom area with relief taken into account) and volume were calculated through the kriging method. Also, at each grid point, substrate type and benthic organisms were identified. Substrate types were categorized as follows: mud (silty substratum), sand (grain size < 1 mm), gravel (grains ≤ 50 mm) and rock (consolidated substrate > 50 mm). Both benthic cover and substrate type were expressed in percentage. The rugosity index was generated by the ratio between surface area and planar area. The height of each tidepool was defined as the vertical distance between pool water surface and sea level at low tide. Subtidal and supralittoral distances were measured using a 30 m metric-tape.

Fish were sampled using hand nets after application of the anesthetic clove oil (40 mg·l^−1^ in ethanol), an efficient and selective method not inducing mortality in non-target fauna^61^. After collection, individuals were frozen at −20 °C and key specimens were sorted and fixed in 10% formaldehyde and later preserved in 70% ethanol. Fish sampling was carried out under approval and accordance with local Ethics Committee (Protocol 29/2016 of the Ethics Committee of Animal Use – Federal University of Espírito Santo). Measurements for total length and wet weight were taken with calipers (0.1 mm) and digital weighing scale (0.01 g). Species were classified in relation to their degree of residency in pools following published works in the areas studied^18,62,63^ as well as underwater observations by the authors. Fishes grouped as Permanent residents can spend their entire life in pools and are frequently highly adapted for intertidal life. Secondary residents or opportunists spend only part of their life-history in tidepools, usually as juveniles. Transients, which are species that only occasionally or accidentally enter in tidepools, generally have no specialized adaptations for intertidal life, and normally occur in large tidepools for a short period of time (from a tidal cycle to several weeks)^19,22,64^. Species also were assigned to main trophic group (i.e., carnivores, herbivores, omnivores and detritivores) or major subcategory (macro-carnivores, mobile invertebrate feeders, omnivores, territorial herbivores and roving herbivores) based on classical and current literature for Atlantic fishes trophic ecology^39,45,65,66^, complemented by *in situ* observations by the authors.

### Data analysis

Differences in tidepool fish assemblages were tested using fish density data (ind·m^−3^) through two-way PERMANOVA based on log-transformed density values of the 95% most-representative species with the factors region (fixed) and site (random, nested in region). PERMANOVA comparisons used a type III sum of squares and 9,999 permutations under the reduced model using a matrix based on Bray-Curtis similarity. Additionally, non-metric multidimensional scaling (nMDS) was used to visualize data dispersion. In the same routine, similarity percentages analysis (SIMPER) was applied to verify what species were the most representative and contribute more to characterize the sites at a level of cumulative contribution of 80% for each site. All analyses above were performed in PRIMER v6^67^. To better evaluate the differences and representativeness of trophic groups in tidepool assemblages, trophic structure was evaluated through ANOSIM to verify dissimilarities between oceanic and coastal sites. In addition, differences in trophic pattern between oceanic and coastal regions were tested through Kruskal-Wallis and Mann-Whitney tests using fish densities (ind·m^−3^) of the main trophic groups^68^.

We used multivariate regression tree (MRT) combined with a principal component analysis of the dependent (fish density) and independent (environmental) variables to predict how explanatory variables influence the density of the most representative intertidal fish species. MRT was performed in R software^69^ through mvpart package^70^ using eighteen environmental explanatory variables, two categorical and sixteen numerical: Region (oceanic and coastal), Substrate type (carbonate and non-carbonate), three variables describing inorganic substrate structure (Sand, Gravel and Rock coverage), three variables depicting the biological cover (Algae, Sessile animal and Turf algae cover), three physicochemical water variables (Water temperature, Salinity and pH) and seven tidepool morphometrical or positional variables (Depth, Rugosity, Volume, Surface area, Pool height, Distance to subtidal and to supralittoral). Prior to analysis, environmental data were standardized to the same mean (with standard deviation of 1) and fish densities were log-transformed. Thus, we built a hierarchical tree to graphically represent the combination of best explanatory variables and indicator species in order to allow deductions about species realized niches^71,72^.

## Electronic supplementary material

Supplementary material

## References

[1] O’Dor, R. *The unknown ocean: the baseline report of the census of marine life research program*. *CORE, Washington* (2003).

[2] Jackson JBC (1997). Reefs since Columbus. Coral Reefs.

[3] Jackson JBC (2001). What was natural in the coastal oceans?. Proceedings of the National Academy of Sciences of the United States of America.

[4] Cardinale BJ (2012). Biodiversity loss and its impact on humanity. Nature.

[5] Halpern BS (2008). A global map of human impact on marine ecosystems. Science.

[6] McCauley DJ (2015). Marine defaunation: Animal loss in the global ocean. Science.

[7] Naeem S, Duffy JE, Zavaleta E (2012). The functions of biological diversity in an age of extinction. Science.

[8] Moberg F, Folke C (1999). Ecological goods and services of coral reef ecosystems. Ecological Economics.

[9] Mumby PJ (2008). Coral reef habitats as surrogates of species, ecological functions, and ecosystem services. Conservation Biology.

[10] Horn, M. M., Martin, K. L. M. & Chotkowski, M. A. *Intertidal fishes: life in two worlds* (Elsevier, 1999).

[11] Bellwood DR, Hughes TP, Folke C, Nyström M (2004). Confronting the coral reef crisis. Nature.

[12] Dulvy NK, Sadovy Y, Reynolds JD (2003). Extinction vulnerability in marine populations. Fish and Fisheries.

[13] Andrades R (2017). Fringe on the brink: Intertidal reefs at risk. Science.

[14] Gibson, R. N. In *Intert*idal f*is*hes: *life in two worlds* (eds Horn, M. M., Martin, K. L. M. & Chotkowski, M. A.) 97–125 (Academic Press, 1999).

[15] White GE, Brown C (2013). Site fidelity and homing behaviour in intertidal fishes. Marine Biology.

[16] Krück NC, Chargulaf CA, Saint-Paul U, Tibbetts IR (2009). Early post-settlement habitat and diet shifts and the nursery function of tidepools during *Sillago* spp. recruitment in Moreton Bay, Australia. Marine Ecology Progress Series.

[17] Dias, M. *et al*. Intertidal pools as alternative nursery habitats for coastal fishes. *Marine Biology Research* 1–14, 10.1080/17451000.2016.1143106 (2016).

[18] Macieira RM, Simon T, Pimentel CR, Joyeux J-C (2015). Isolation and speciation of tidepool fishes as a consequence of Quaternary sea-level fluctuations. Environmental Biology of Fishes.

[19] Gibson, R. N. & Yoshiyama, R. M. In *Int**ertidal**f**is**hes*: *life in two worlds* (eds Horn, M. M., Martin, K. L. M. & Chotkowski, M. A.) 264–296 (Academic Press, 1999).

[20] DeMartini, E. E. In *Int*ertidal f*is*hes: *life in two worlds* (eds Horn, M. H., Martin, K. L. M. & Chotkowski, M. A.) 143–164 (Academic Press, 1999).

[21] Zander, C., Nieder, J. & Martin, K. In *Intertidal fishes: life in two worlds* (eds Horn, M. M., Martin, K. L. M. & Chotkowski, M. A.) 26–53, 10.1016/B978-012374473-9.00017-5 (Academic Press, 1999).

[22] Griffiths SP (2003). Rockpool ichthyofaunas of temperate Australia: species composition, residency and biogeographic patterns. Estuarine, Coastal and Shelf Science.

[23] Sorte CJB, Bracken MES (2015). Warming and elevated CO2 interact to drive rapid shifts in marine community production. PloS One.

[24] Alexander TJ, Gladstone W (2013). Assessing the effectiveness of a long-standing rocky intertidal protected area and its contribution to the regional conservation of species, habitats and assemblages. Aquatic Conservation: Marine and Freshwater Ecosystems.

[25] Goatley CHR, González-Cabello A, Bellwood DR (2016). Reef-scale partitioning of cryptobenthic fish assemblages across the Great Barrier Reef, Australia. Marine Ecology Progress Series.

[26] Willis T (2001). Visual census methods underestimate density and diversity of cryptic reef fishes. Journal of Fish Biology.

[27] Cox TE, Baumgartner E, Philippoff J, Boyle KS (2011). Spatial and vertical patterns in the tidepool fish assemblage on the island of O’ahu. Environmental Biology of Fishes.

[28] White, G. E., Hose, G. C. & Brown, C. Influence of rock-pool characteristics on the distribution and abundance of inter-tidal fishes. *Marine Ecology* 1–13, 10.1111/maec.12232 (2014).

[29] Schemske DW, Mittelbach GG, Cornell HV, Sobel JM, Roy K (2009). Is there a latitudinal gradient in the importance of biotic interactions?. Annual Review of Ecology, Evolution, and Systematics.

[30] Arakaki S, Tsuchiya M, Tokeshi M (2014). Testing latitudinal patterns of tidepool fish assemblages: local substrate characteristics affect regional-scale trends. Hydrobiologia.

[31] Arakaki S, Tokeshi M (2006). Short-term dynamics of tidepool fish community: diel and seasonal variation. Environmental Biology of Fishes.

[32] Davis JLD (2001). Diel changes in habitat use by two tidepool fishes. Copeia.

[33] Rojas JM, Ojeda FP (2010). Spatial distribution of intertidal fishes: a pattern dependent on body size and predation risk?. Environmental Biology of Fishes.

[34] Fernández-Palacios JM (2016). Island biogeography: Shaped by sea-level shifts. Nature.

[35] Helmus MR, Mahler DL, Losos JB (2014). Island biogeography of the Anthropocene. Nature.

[36] Cameron RAD (2013). Snails on oceanic islands: Testing the general dynamic model of oceanic island biogeography using linear mixed effect models. Journal of Biogeography.

[37] DeMartini EE, Friedlander AM (2004). Spatial patterns of endemism in shallow-water reef fish populations of the Northwestern Hawaiian Islands. Marine Ecology Progress Series.

[38] White GE, Brown C (2014). A comparison of spatial learning and memory capabilities in intertidal gobies. Behavioral Ecology and Sociobiology.

[39] Ferreira CEL, Floeter SR, Gasparini JL, Ferreira BP, Joyeux JC (2004). Trophic structure patterns of Brazilian reef fishes: a latitudinal comparison. Journal of Biogeography.

[40] Longo GO (2015). Between-habitat variation of benthic cover, reef fish assemblage and feeding pressure on the benthos at the only atoll in South Atlantic: Rocas Atoll, NE Brazil. PloS One.

[41] Krajewski JP, Floeter SR (2011). Reef fish community structure of the Fernando de Noronha Archipelago (Equatorial Western Atlantic): The influence of exposure and benthic composition. Environmental Biology of Fishes.

[42] Pinheiro HT, Ferreira CEL, Joyeux JC, Santos RG, Horta PA (2011). Reef fish structure and distribution in a south-western Atlantic Ocean tropical island. Journal of Fish Biology.

[43] Pillai CSG, Gopakumar G, Mohan M (1992). Ichthyofauna of the intertidal reef flats of Minicoy Atoll, Lakshadweep: An analysis of its structure, relative abundance and food. Journal of the Marine Biological Association of India.

[44] Norton, S. F. & Cook, A. E. In Inter*tid*al f*is*hes: *life in two worlds* (eds Horn, M. H., Martin, K. L. M. & Chotkowski, M. A.) 223–263 (Academic Press, 1999).

[45] Pimentel, C. R., Soares, L. S. H., Macieira, R. M. & Joyeux, J.-C. Trophic relationships in tidepool fish assemblages of the tropical Southwestern Atlantic. *Marine Ecology* e12496, 10.1111/maec.12496 (2018).

[46] Martin, K. L. M. & Bridges, C. R. In In*ter*tidal f*is*hes: *life in two worlds* (eds Horn, M. M., Martin, K. L. M. & Chotkowski, M. A.) 54–78 (Academic Press, 1999).

[47] Andrades R, Macieira RM, Reis-Filho JA, Giarrizzo T, Joyeux J-C (2016). Trapped in their own ‘home’: unexpected records of intertidal fish desiccation during low tides. Journal of Applied Ichthyology.

[48] Ord TJ, Cooke GM (2016). Repeated evolution of amphibious behavior in fish and its implications for the colonization of novel environments. Evolution.

[49] Brown CR, Gordon MS, Chin HG (1991). Field and laboratory observations on microhabitat selection in the amphibious red sea rockskipper fish, *Alticus kirki* (Family Blenniidae). Marine Behaviour and Physiology.

[50] Martin KL (2014). Theme and variations: amphibious air-breathing intertidal fishes. Journal of fish biology.

[51] Ikebe Y, Oishi T (1997). Relationships between environmental factors and diel and annual changes of the behaviors during low tides in *Periophthalmus modestus*. Zoological Science.

[52] Braithwaite VA, De Perera TB (2006). Short-range orientation in fish: How fish map space. Marine and Freshwater Behaviour and Physiology.

[53] Pinheiro HT (2017). Island biogeography of marine organisms. Nature.

[54] Pinheiro HT (2015). Fish biodiversity of the Vitória-Trindade seamount chain, southwestern Atlantic: An updated database. PloS One.

[55] Labelle M, Nursall JR (1992). Population biology of the redlip blenny *Ophioblennius atlanticus* (Sylvester) in Barbados. Bulletin of Marine Science.

[56] Beldade R, Pedro T, Gonçalves EJ (2007). Pelagic larval duration of 10 temperate cryptobenthic fishes. Journal of Fish Biology.

[57] Lubbock R, Edwards A (1981). The fishes of Saint Paul’s Rocks. Journal of Fish Biology.

[58] Luiz OJ (2015). Community structure of reef fishes on a remote oceanic island (St Peter and St Paul’s Archipelago, equatorial Atlantic): The relative influence of abiotic and biotic variable. s. Marine and Freshwater Research.

[59] Willis TJ, Roberts CD (1996). Recolonisation and recruitment of fishes to intertidal rockpools at Wellington, New Zealand. Environmental Biology of Fishes.

[60] Paulin, C. D. & Roberts, C. D. *Biogeography of New Zealand rockpool fishes*. *Proceedings of the Second International Temperate Reef Symposium* (NIWA Marine, Wellington, 1993).

[61] Griffiths S (2000). The use of clove oil as an anaesthetic and method for sampling intertidal rockpool fishes. Journal of Fish Biology.

[62] Macieira RM, Joyeux J-C (2011). Distribution patterns of tidepool fishes on a tropical flat reef. Fishery Bulletin.

[63] Oliveira RRS, Macieira RM, Giarrizzo T (2016). Ontogenetic shifts in fishes between vegetated and unvegetated tidepools: assessing the effect of physical structure on fish habitat selection. Journal of Fish Biology.

[64] Barlow, G. W. In *Behaviour and conservation of littoral fishes* (eds. Almada, V. C., Oliveira, R. F. & Gonçalves, E. J.) 3–32 (ISPA, 1999).

[65] Randall JE (1967). Food habits of reef fishes of the West Indies. Studies in Tropical Oceanography.

[66] Zamprogno, C. Distribuição e hábitos alimentares dos peixes na zona entremarés de recifes rochosos da praia de Manguinhos, Espírito Santo. (MSc dissertation, Universidade Estadual de Campinas, Campinas, Brazil, 1989).

[67] Clarke, K. R. & Gorley, R. N. *PRIMERv6: User Manual/Tutorial*. (2006).

[68] Zar, J. H. *Biostatistical analysis*. (Prentice Hall, Inc., 2010).

[69] R Core Team. R: A language and environment for statistical computing. R Foundation for Statistical Computing, Vienna, Austria. www.R-project.org/.

[70] Therneau, T. M., Atkinson, B., Ripley, B., Oksanen, J. & De’ath, G. mvpart: Multivariate partitioning (2014).

[71] De’ath G, Fabricius KE (2000). Classification and regression trees: a powerful yet simple technique for ecological data analysis. Ecology.

[72] De’ath G (2002). Multivariate regression trees: a new technique for modeling species-environment relationships. Ecology.

[73] Hachich NF (2015). Island biogeography: patterns of marine shallow-water organisms in the Atlantic Ocean. Journal of Biogeography.

