# Supplementary material for: Endemic fish species structuring oceanic intertidal reef assemblages
Source: Sci Rep. 2018 Jul 17;8:10791. doi: 10.1038/s41598-018-29088-0 (PMC6050318; doi:10.1038/s41598-018-29088-0)
Supplement: Supplementary file 1 — Supplementary material [file 41598_2018_29088_MOESM1_ESM.pdf]

Endemic fish species structuring oceanic intertidal reef assemblages

Andrades et al.

Supplementary Material

SM1- Brief description of studied areas in the southwestern Atlantic Ocean.
SM2 – Supplementary Table 1: Intertidal reef fish species sampled in the present study.
SM2 – Supplementary Table 2: SIMPER cumulative contribution (>80%) of island and Brazilian coastal endemic species densities.
Supplementary Figure S1. Intertidal reefs of oceanic and coastal sites

Andrades et al. Supplementary Material

Methods

Studied sites in the southwestern Atlantic

Oceanic sites

Rocas Atoll (03°51' S; 33°49' W) is the only atoll in the South Atlantic, distant 266 km of the mainland (state of Rio Grande do Norte). It has an elliptical shape and is primarily built by coralline algae, vermetid gastropods and hermatypic corals. Tidal regime is semidiurnal with mesotides reaching 3.8 m. At high tide, water almost covers the entire atoll, including all tidepools [1]. Climate is tropical with water temperature and salinities values in tidepools ranging 28-34°C and 3539, respectively (present study). Rocas Atoll probably is one of the most near-pristine areas of the Southwestern Atlantic being the first Marine Reserve established in Brazil (1978). However, effective monitoring and enforcement only began in 1991 [2]. No public visitation is allowed.

Fernando de Noronha Archipelago (03°50' S; 32°25' W) is located 345 km from the continent (state of Rio Grande do Norte) and belongs to the same volcanic ridge than Rocas Atoll. The study was conducted on the main island, Fernando de Noronha, where phonolite intertidal reefs often occur associated with biogenic carbonate substrate composed by encrusting coralline algae. Tidal regime is semidiurnal with mesotides (3.2 m maximum). Climate is similar to that of Rocas with temperature around 25°C and 27°C in air and water, respectively [3]. In tidepools, temperature varied 27-35°C and salinity 35-40 (present study). Most of Fernando de Noronha and surrounding waters are included in a National Marine Park created in 1988, ensuring some protection from fishing activities and control over tourism. In 2001, Noronha and Rocas were declared a World Heritage Site by UNESCO (United Nations Educational, Scientific and Cultural Organization) due to their role as oases to marine wildlife and the presence of endemic and endangered seabirds, turtles and reef fish species, among others [4].

Trindade Island (20°30' S; 29°20' W) is tropical and located 1,160 km off the coast of the state of Espírito Santo; it is the most isolated insular environment of the Brazilian Province[5]. Trindade and, about 40km farther east, Martin Vaz Archipelago are the only emerged sites and the eastern end of the Vitória-Trindade submarine chain (VTC). The VTC comprises a set of 17

volcanic seamounts, sheltering rich fish fauna and benthic reef habitats, that have functioned as stepping-stones and allowed some connectivity between reef populations along the VTC [6,7]. Among Brazilian oceanic islands, Trindade has the highest reef fish endemism rate (9.6%; Pinheiro et al. 2015), while being the youngest island (3.7 My; the Noronha-Rocas complex is about 12.3 My-old and Saint Peter and Saint Paul's Archipelago about 9 My-old; Ferrari & Riccomini 1999, Hekinian et al. 2000, Castro 2009). Intertidal habitats of Trindade are made of biogenic carbonate (mostly encrusting coralline algae) and phonolite rocks substrates under semidiurnal microtides reaching 1.7 m [10]. In our study, temperature in tidepools varied 27-34°C and salinities 35-41. Since 1957, the Brazilian Navy occupies the island and it maintains an oceanographic station (POIT – Posto Oceanográfico da Ilha da Trindade). Public visitation of the islands is restricted to research and military purposes, but commercial and recreational fishing is allowed.

Coastal sites

Salinópolis (00°36' S; 47°21' W) is located in the Amazonian region of Brazil (state of Pará), but the influence of the Amazon and other large rivers is moderate in contrast to the direct influence of medium and small estuaries distributed along the Pará coastline. In Salinópolis, the intertidal is mostly inserted into a carbonate reef derived from the Pirabas formation (early Miocene) and composed mainly of fossil coralline algae, corals, sponges and bryozoans [11]. The reef is isolated in a large sandy area, tidepools show a relatively low coverage of macroalgae and temperature and salinity ranged 31-35°C and 36-41, respectively (present study). Tidal regime is of semidiurnal macrotides reaching over 5 m. Salinópolis is the main touristic beach-spot on the Pará coastline, and human-related impacts on the beach such as organism removal and reef trampling are expected to be high, especially during summer vacations [12].

Jericoacoara beach (02°47' S; 40°30' W) is situated in northeastern Brazil, receives little riverine influence and is governed by semidiurnal mesotides (4 m). Quartzite beach rocks with low complexity tidepools covered by dense fleshy macroalgae dominated the intertidal. Seasonally, harsh erosional events driven by sand-dune sediment occur, changing the intertidal-scape covering and uncovering tidepools. Temperature and salinity in tidepools varied 29-32°C and 39-41, respectively (present study). The beach is inserted into Jericoacoara National Park. Created in 2002, the area of *ca*. 9,000 hectares comprises sand dunes, mangrove areas, subtidal and intertidal shores open to public visitation.

Anchieta is a city in the state of Espírito Santo, southeastern Brazil, which shelters Castelhanos beach (20°49' S; 40°36' W), a tropical carbonatic flat reef composed mainly by encrusting coralline algae and stony coral skeletons [13]. Fleshy macroalgae and sessile animals (sea

urchins and zoanthids) are abundant in tidepools. Water temperature and salinity varied 24-35°C and 36-41, respectively. Tidal regime is semidiurnal microtides reaching 1.8 m [13]. Similar to aforementioned coastal beaches, Castelhanos is touristic and suffers human-related impacts such as reef trampling, organism removal and fishing.

References

1. Gherardi, D. F. M. & Bosence, D. W. J. Composition and community structure of the coralline algal reefs from Atol das Rocas, South Atlantic, Brazil. *Coral Reefs* **19,** 205–219 (2001).
2. Longo, G. O. *et al.* Between-habitat variation of benthic cover, reef fish assemblage and feeding pressure on the benthos at the only atoll in South Atlantic: Rocas Atoll, NE Brazil. *PloS One* **10,** e0127176 (2015).
3. Castro, J. in *Ilhas Oceânicas Brasileiras: da pesquisa ao manejo* (eds. Mohr, L., Castro, J., Cosata, P. & Alves, R. J. V.) 33–53 (Ministério do Meio Ambiente - Secretaria de Biodiversidade e Florestas, 2009).
4. UNESCO. World Heritage List. *World Heritage Site* (2017). Available at: http://whc.unesco.org/en/list/1000.
5. Briggs, J. C. & Bowen, B. W. A realignment of marine biogeographic provinces with particular reference to fish distributions. *Journal of Biogeography* **39,** 12–30 (2012).
6. Pinheiro, H. T., Joyeux, J.-C. & Moura, R. L. Reef oases in a seamount chain in the southwestern Atlantic. *Coral Reefs* 2014 (2014). doi:10.1007/s00338-014-1211-9
7. Pinheiro, H. T. *et al.* Fish biodiversity of the Vitória-Trindade seamount chain, southwestern Atlantic: An updated database. *PloS One* **10,** e0118180 (2015).
8. Ferrari, A. L. & Riccomini, C. Campo de esforços Plio-Pleistocênico na Ilha da Trindade (Oceano Atlântico Sul, Brasil) e sua relação com a tectônica regional. *Revista Brasileira de Geociências* **29,** 195–202 (1999).
9. Hekinian, R. *et al.* Submersible observations of equatorial atlantic mantle: The St. Paul Fracture Zone region. *Marine Geophysical Researches* **21,** 529–560 (2000).
10. Macieira, R. M., Simon, T., Pimentel, C. R. & Joyeux, J.-C. Isolation and speciation of tidepool fishes as a consequence of Quaternary sea-level fluctuations. *Environmental Biology of Fishes* **98,** 385–393 (2015).
11. Távora, V. A., Neto, I. L. N. & Maciel, L. M. Geology and paleontology of the reef facies of the Pirabas Formation (Early Miocene). *Revista do Instituto de Geociências - USP* **13,** 23–40

(2013).

12. Adrião, D. Dream catchers: labor relations and social change among fisherman families due to summer vacations and beach tourism in Salinópolis, Pará. *Boletim do Museu Paraense Emílio Goeldi. Ciências Humanas* **1,** 11–21 (2006).

13. Macieira, R. M. & Joyeux, J.-C. Distribution patterns of tidepool fishes on a tropical flat reef. *Fishery Bulletin* **109,** 305–315 (2011).

Supplementary Material

SM2- Table 1

Supplementary Table S1. Family and species list, fish density (individuals per m^3), total length (TL mm) and range (maximum and minimum), main trophic category, feeding habits (MCAR = macro-carnivore, MINV = mobile invertebrate feeder, OMNI = omnivore, DETR = detritivore, THER = territorial herbivore and RHER = roving herbivore) and residency status. Details about literature used to categorize trophic groups, feeding habits and residency status are provided in Material and Methods section.

**Family** / Species	**Density (±SE)**		**TL range**	**Trophic category**	**Feeding habit**	**Residency status**
	**Oceanic**	**Coastal**				
**Ginglymostomatidae**						
Ginglymostoma cirratum (Bonnaterre 1788)	0.1	—	28	Carnivore	MCAR	Secondary resident
**Muraenidade**						
Gymnothorax funebris Ranzani 1840	—	1 (0.6)	45 – 649	Carnivore	MCAR	Permanent resident
Echidna catenata (Bloch 1795)	0.7 (0.5)	—	296 – 365	Carnivore	MCAR	Permanent resident
Enchelycore nigricans (Bonnaterre 1788)	0.7 (0.7)	—	243 – 762	Carnivore	MCAR	Permanent resident
**Ophichthidae**						
Myrichthys breviceps (Richardson 1848)	0.047		752	Carnivore	MCAR	Secondary resident
**Holocentridae**						
Holocentrus adscencionis (Osbeck 1765)	1.4 (0.9)	—	116 – 131	Carnivore	MINV	Secondary resident
**Batrachoididae**						
Thalassophryne nattereri Steindachner 1876	—	0.2 (0.1)	100 – 123	Carnivore	MCAR	Secondary resident
**Gobiidae**						
Undescribed *Barbulifer* sp.	—	4.7 (2.5)	11 – 30	Carnivore	MINV	Permanent resident
Bathygobius brasiliensis Carvalho-Filho & De Araújo, 2017 ††	69.5 (26.3)	—	8 – 97	Carnivore	MINV	Permanent resident
Bathygobius geminatus Tornabene, Baldwin and Pezold 2010	—	133.2 (65.3)	13 – 64	Carnivore	MINV	Permanent resident
Bathygobius soporator (Valenciennes 1837)	—	18.6 (7.5)	20 – 106	Carnivore	MINV	Permanent resident
Undescribed *Coryphopterus* sp. ††	0.3 (0.3)	—	26 – 43	Carnivore	MINV	Secondary resident
Coryphopterus glaucofrenum Gill 1863	—	0.2 (0.2)	27 – 39	Carnivore	MINV	Permanent resident
Ctenogobius boleosoma (Jordan & Gilbert 1882)	—	0.2	13	Carnivore	MINV	Permanent resident
Gnatholepis thompsoni Jordan 1904	1.1 (0.7)	—	22 – 35	Omnivore	OMNI	Secondary resident
Gobiosoma alfiei Joyeux & Macieira 2015	—	0.5 (0.4)	18 – 20	Carnivore	MINV	Permanent resident
Undescribed *Gobiosoma* sp.	—	4.4 (2.3)	15 – 36	Carnivore	MINV	Permanent resident
Undescribed *Lythrypnus* sp. ††	2.7 (1.6)	—	11 – 21	Carnivore	MINV	Secondary resident
Priolepis dawsoni Greenfield 1989	1.9 (1.7)	—	13 – 19	Carnivore	MINV	Secondary resident
**Pomacentridae**						

Abudefduf saxatilis (Linnaeus 1758)	10 (1.7)	4.8 (2.1)	16 – 96	Omnivore	OMNI	Secondary resident
Stegastes fuscus (Cuvier 1830)	—	0.8 (0.4)	21 – 90	Herbivore	THER	Permanent resident
Stegastes fuscus trindadensis (Cuvier 1830) †	12.7 (7.2)	—	14 – 95	Herbivore	THER	Permanent resident
Stegastes pictus (Castelnau 1855)	0.5	—	35	Herbivore	THER	Transient
Stegastes rocasensis (Emery 1972) ††	36.2 (12.9)	—	15 – 98	Herbivore	THER	Permanent resident
Stegastes variabilis (Castelnau 1855)	—	0.1	41	Herbivore	THER	Secondary resident
**Mugilidae**						
Mugil curema Valenciennes 1836	—	6.4 (5.8)	26 – 42	Detritivore	DETR	Transient
Mugil curvidens Valenciennes 1836	1.1 (1)	—	44 – 80	Detritivore	DETR	Transient
**Dactyloscopidae**						
Storrsia olsoni Dawson 1982 ††	0.8 (0.6)	—	22 – 29	Carnivore	MINV	Permanent resident
**Blenniidae**						
Entomacrodus vomerinus (Valenciennes 1836)	327.2 (150)	—	12 – 106	Herbivore	THER	Permanent resident
Undescribed *Entomacrodus* sp. †	194.3 (87.6)	—	20 – 69	Herbivore	THER	Permanent resident
Hypleurochilus fissicornis (Quoy & Gaimard 1824)	—	2.7 (2.3)	19 – 51	Carnivore	MINV	Permanent resident
Omobranchus punctatus (Valenciennes 1836)	—	12.2 (7)	32 – 72	Herbivore	THER	Permanent resident
Ophioblennius trinitatis Miranda Ribeiro 1919	45.2 (14.3)	—	46 – 103	Herbivore	THER	Permanent resident
Scartella cristata (Linnaeus 1758)	—	53.6 (16.1)	12 – 93	Herbivore	THER	Permanent resident
Scartella itajobi Rangel & Mendes 2009 ††	60 (19.6)	—	13 – 67	Herbivore	THER	Permanent resident
Scartella poiti Rangel, Gasparini & Guimarães 2004 †	6.6 (5.9)	—	40 – 72	Herbivore	THER	Permanent resident
**Labrisomidae**						
Labrisomus conditus Sazima, Carvalho-Filho, Gasparini & Sazima 2009 ††	0.5 (0.4)	—	69 – 144	Carnivore	MCAR	Secondary resident
Gobioclinus kalisherae (Jordan 1904)	0.2 (0.2)	—	38 – 72	Carnivore	MCAR	Secondary resident
Labrisomus nuchipinnis (Quoy & Gaimard 1824)	5.3 (2.2)	4.7 (1.5)	35 – 158	Carnivore	MCAR	Permanent resident
Undescribed *Malacoctenus* sp. ††	4.9 (3.2)	—	23 – 53	Carnivore	MINV	Permanent resident
Malacoctenus brunoi Guimarães, Nunan & Gasparini 2010 †	9.3 (4.4)	—	31 – 52	Carnivore	MINV	Permanent resident
Malacoctenus delalandii (Valenciennes 1836)	—	12.2 (3.9)	18 – 59	Carnivore	MINV	Permanent resident
Paraclinus arcanus Guimarães & Barcellar 2002	—	0.1	23	Carnivore	MINV	Permanent resident
Starksia multilepis Williams & Mounts 2003 ††	7.7 (3.1)	—	12 – 32	Carnivore	MINV	Permanent resident
**Gobiesocidae**						
Gobiesox barbatulus Starks 1913	—	0.5 (0.3)	21 – 36	Carnivore	MINV	Secondary resident
Undescribed *Tomicodon* sp. ††	1 (0.7)	—	20 – 23	Carnivore	MINV	Permanent resident
Undescribed *Tomicodon* sp. †	11.7 (9.3)	—	10 – 21	Carnivore	MINV	Permanent resident
**Labridae**						

Halichoeres penrosei Starks 1913	6.7 (3.9)	—	18 – 90	Carnivore	MINV	Secondary resident
Halichoeres poeyi (Steindachner 1867)	—	0.9 (0.8)	64 – 84	Carnivore	MINV	Secondary resident
Sparisoma axillare (Steindachner 1878)	—	0.4 (0.2)	39 – 78	Herbivore	RHER	Secondary resident
Sparisoma frondosum (Agassiz 1831)	0.2	—	35	Herbivore	RHER	Secondary resident
Thalassoma noronhanum (Boulenger 1890)	18.4 (5.6)	—	16 – 72	Carnivore	MINV	Secondary resident
**Epinephelidae**						
Cephalopholis fulva (Linnaeus 1758)	0.1 (0.1)	—	58	Carnivore	MCAR	Transient
**Haemulidae**						
Genyatremus luteus (Bloch 1790)	—	2.4 (1.4)	17 – 99	Carnivore	MINV	Transient
**Lutjanidae**						
Lutjanus jocu (Bloch & Schneider 1801)	—	0.6 (0.3)	21 – 32	Carnivore	MCAR	Secondary resident
**Scorpaenidae**						
Scorpaena plumieri Bloch 1789	—	0.4 (0.2)	49 – 71	Carnivore	MCAR	Secondary resident
**Acanthuridae**						
Acanthurus bahianus Castelnau 1855	—	2.6 (1.3)	35 – 43	Herbivore	RHER	Secondary resident
Acanthurus chirurgus (Bloch 1787)	1.6 (1.2)	0.4 (0.3)	33 – 68	Herbivore	RHER	Secondary resident
**Tetraodontidae**						
Sphoeroides greeleyi (Gilbert 1900)	—	0.3	102	Carnivore	MINV	Transient

† Species endemics to Trindade-Martin Vaz insular complex, †† Species endemics to Rocas-Noronha insular complex.

Supplementary Material S2

Supplementary Table S2. SIMPER cumulative contribution (>80%) of island and Brazilian coastal endemic species densities for studied island and coastal intertidal assemblages, respectively. Endemic important species for Salinópolis were *Barbulifer* sp. (32%) and *Gobiosoma* sp. (10%).

Group	Oceanic (%)			Coastal (%)		
	Rocas	Noronha	Trindade	Salinópolis	Jericoacoara	Anchieta
Endemic	52	44	65	42	0	0
Non-endemic	29	41	19	44	80	84

Supplementary Figure

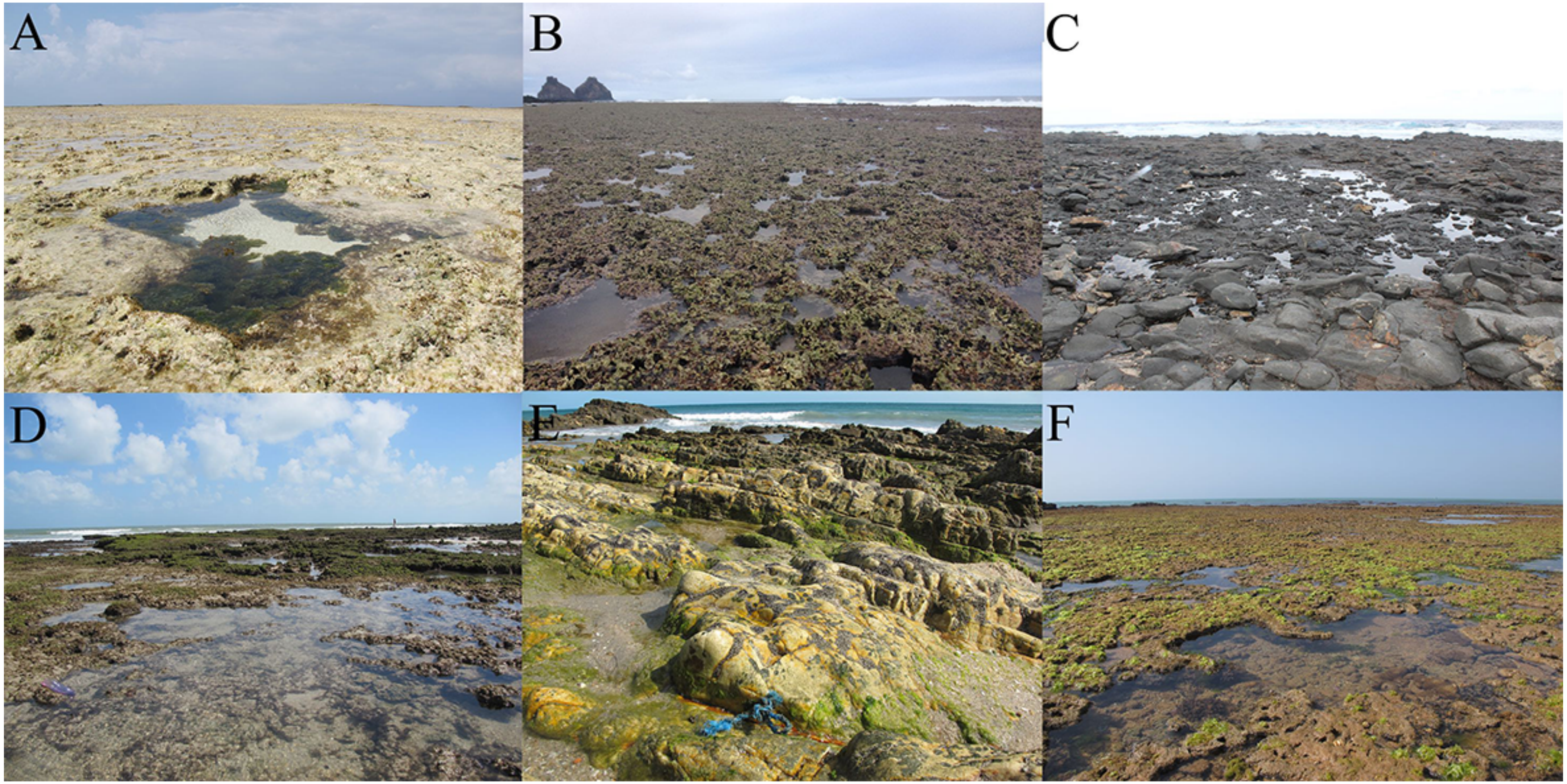

Supplementary Figure S1. Intertidal reefs of oceanic islands Rocas Atoll (A), Fernando de Noronha (B) and Trindade Island (C), and coastal sites Salinópolis (D), Jericoacoara (E) and Anchieta (F).
